# Sex differences in central insulin action: Effect of intranasal insulin on neural food cue reactivity in adults with normal weight and overweight

**DOI:** 10.1038/s41366-022-01167-3

**Published:** 2022-06-17

**Authors:** Lore Wagner, Ralf Veit, Louise Fritsche, Hans-Ulrich Häring, Andreas Fritsche, Andreas L. Birkenfeld, Martin Heni, Hubert Preissl, Stephanie Kullmann

**Affiliations:** 1 Institute for Diabetes Research and Metabolic Diseases of the Helmholtz Center Munich at the University of Tübingen, Tübingen, Germany; 2 German Center for Diabetes Research (DZD e.V.), Tübingen, Germany; 3 Department of Internal Medicine, Division of Endocrinology, Diabetology and Nephrology, Eberhard Karls University Tübingen, Tübingen, Germany; 4 Nutritional and Preventive Medicine, Eberhard Karls University Tübingen, Tübingen, Germany; 5 Institute for Clinical Chemistry and Pathobiochemistry, University Hospital Tübingen, Tübingen, Germany; 6 Department of Internal Medicine I, Division of Endocrinology and Diabetology, Ulm University Hospital, Ulm, Germany; 7 Institute for Diabetes and Obesity, Helmholtz Diabetes Center, Helmholtz Center Munich, German Research Center for Environmental Health (GmbH), Neuherberg, Germany

**Keywords:** Obesity, Neuroscience

## Abstract

**Background/Objectives:**

Central insulin action influences cognitive processes, peripheral metabolism, and eating behavior. However, the contribution of obesity and sex on central insulin-mediated neural food cue processing still remains unclear.

**Subjects/Methods:**

In a randomized within-participant design, including two visits, 60 participants (30 women, BMI 18–32 kg/m^2^, age 21–69 years) underwent a functional MRI task measuring blood oxygen level-dependent (BOLD) signal in response to visual food cues after intranasal insulin or placebo spray administration. Central insulin action was defined as the neural BOLD response to food cues after insulin compared to placebo administration. Afterwards, participants were asked to rate the food cues for desire to eat (i.e., wanting rating). For statistical analyses, participants were grouped according to BMI and sex.

**Results:**

Food cue reactivity in the amygdala showed higher BOLD activation in response to central insulin compared to placebo. Furthermore, women with overweight and obesity and men of normal weight showed higher BOLD neural food cue responsivity to central insulin compared to placebo. Higher central insulin action in the insular cortex was associated with better peripheral insulin sensitivity and higher cognitive control. Moreover, central insulin action in the dorsolateral prefrontal cortex (DLPFC) revealed significant sex differences. In response to central insulin compared to placebo, men showed lower DLPFC BOLD activity, whereas women showed higher DLPFC activity in response to highly desired food cues. On behavioral level, central insulin action significantly reduced hunger, whereas the desire to eat, especially for low caloric food cues was significantly higher with central insulin than with placebo.

**Conclusions:**

Obesity and sex influenced the central insulin-mediated neural BOLD activity to visual food cues in brain regions implicated in reward and cognitive control. These findings show that central insulin action regulates food response differentially in men and women, which may have consequences for metabolism and eating behavior.

## Introduction

After the discovery and isolation of the hormone insulin, 100 years ago, the seminal role of insulin in the periphery was quickly recognized. Scientific interests only later turned to the role of the brain in insulin signaling [1–3]. Since then, evidence is rapidly accumulating that central insulin action plays a vital role in metabolic and cognitive health, including memory, mood and olfaction, eating behavior, and also peripheral metabolism (for review see: [4–6]).

Central insulin action can be assessed by intranasal application, -the delivery of the hormone with a spray through the nose to the brain. Combined with imaging techniques like functional magnetic resonance imaging (fMRI), this allows to study insulin action in the brain non-invasively in humans [6]. Several studies, using intranasal insulin, demonstrated changes in regional resting-state activity and functional connectivity in the hypothalamus, striatum, hippocampus, amygdala, insula, and parts of the prefrontal cortex [7–13]. These are all regions part of an interconnected network regulating eating behavior, which are responsive to a meal, postprandial hormones, and to the taste and sight of food [14]. People with obesity show higher food cue reactivity (FCR), particularly in regions important for emotion and reward regulation, including the insula, amygdala, and orbitofrontal cortex [15] and FCR is even predictive for the outcome of weight-loss interventions (e.g., [16]). In response to central insulin, persons with obesity showed altered activity in reward-related brain regions with subsequent effects on eating behavior-related measures (e.g., failure to reduce food craving and hunger) [8, 11, 12]. A possible explanation could be the role of central insulin action on dopamine signaling. Recent findings demonstrated that intranasal insulin administration directly modulated striatal dopamine levels and functional connectivity of reward pathways in healthy humans [9, 11, 12] and directly modulates dopamine function in the midbrain and nucleus accumbens in animal models [17]. This leads to the assumption, that central insulin action is not only implicated in the regulation of energy homeostasis, but also in reward processing.

Sex also plays a prominent role in appetite regulation and FCR. Women, compared to men, displayed higher activity in frontal (PFC) and reward areas, including striatum and insula in response to high-caloric cues [18] and higher FCR in reward areas was a predictor for BMI in women [19]. Following intranasal insulin administration, a reduction of food intake [20, 21] and food craving [8] as well as slight reductions in body weight and adipose mass [22] were observed in men. In women with normal weight [23] and obesity [24], central insulin action decreased palatable food intake (i.e., cookies) in the postprandial state. Hence, first evidence points to sex-specific effects of central insulin action on eating behavior and appetite regulation.

However, no study thus far has evaluated whether obesity and sex determine central insulin effects on neural FCR. Therefore, our primary aim was to elucidate the effect of central insulin action on appetite and reward regulation by using an FCR task during fMRI in healthy volunteers. Central insulin action was probed by nasal insulin application compared to placebo. We hypothesized a stronger insulin effect on FCR in participants with normal weight compared to participants with overweight and obesity in brain regions involved in eating behavior. Furthermore, we expected sex-dependent effects on FCR in response to central insulin with increased activity in reward-related areas in women with overweight and obesity. On a behavioral level, we hypothesized that central insulin action results in a reduction in perceived hunger and wanting for high-caloric food, particularly in men with normal weight. Furthermore, we intended to explore whether central insulin response on FCR is associated with behavioral and peripheral measures.

## Methods

### Subjects

Seventy participants were recruited for the study. Five participants had to be excluded from the analysis due to incomplete fMRI measurements (technical issues), three based on insufficient data quality (e.g., excessive movement (>2 mm or 2°) or participant fell asleep during the fMRI measurement), one participant was not in a fasted state and one participant had major anatomical abnormalities of the brain.

Datasets of sixty participants (30 women) were used for the final analysis: 37 participants with normal weight (NW group, 20 women, body mass index (BMI) range 18–25 kg/m^2^, and age range 21–69 years), 23 participants with overweight and obesity (OW group, 10 women, BMI range 25–32 kg/m^2^, and age range 24–65 years) (Table 1: shows complete descriptive and metabolic data).

**Table 1:** Participants’ characteristics.

	Normal weight (NW)		Overweight/obesity (OW)		*p*-value
	women	men	women	men	
**N**	20	17	10	13	–
**Age [years]**	42.55 (3.41)	40.29 (3.99)	42.9 (5.35)	47.08 (3.9)	0.514
**BMI [kg/m**^**2**^**]**	22.9 (0.25)	22.66 (0.49)	27.5 (0.7)	27.01 (0.5)	<0.001
**Fasting glucose [mmol/l]**	4.96 (0.09)	5.12 (0.11)	4.95 (0.18)	5.09 (0.09)	0.415
**Fasting insulin [pmol/l]**	62.9 (4.66)	50.65 (5.71)	95.3 (37.26)	70 (8.9)	0.151
**Body fat [%]**	30.9 (0.96)	16.4 (0.93)	38.23 (1.09)	18.82 (1.04)	<0.001
**Insulin sensitivity, OGTT-derived [AU] (ISI**_**Matsuda**_**)**	16.41 (1.6)	18.9 5 (3.13)	18.84 (4.78)	14.78 (2.37)	0.715
**HbA1c [mmol/mol]**	35.25 (0.62)	34.24 (0.69)	36.2 (0.83)	36.92 (1.14)	0.175
**HbA1c [%]**	5.39 (0.06)	5.29 (0.06)	5.48 (0.07)	5.52 (0.1)	0.162

Values in the table given as mean (SEM).

*p*-values: non-parametrical Kruskal-Wallis-H-Test between the 4 groups.

*BMI* Body mass index, *ISI*_*Matsuda*_ Matsuda peripheral insulin sensitivity index.

Participants signed a written informed consent before participation and the study was approved by the local ethics committee of the medical faculty of the University of Tübingen. The study was registered as a clinical trial (NCT04372849).

### Power calculation

In order to evaluate the effect of intranasal insulin versus placebo on neural FCR, we used medium effect size to calculate a total sample size of *n* = 60 using ANOVA repeated measures including within and between interactions (G*Power 3.1.9, α = 0.05, power = 0.95). In previous studies between group differences based on BMI showed large effect sizes (Eta-squared of greater 0.2) for differences in insulin action in the prefrontal cortex [8, 25].

### Experimental design and procedure

Prior to the experiment, all participants underwent a medical examination to assure that they did not suffer from psychiatric, neurological nor metabolic diseases or taking any kind of medication other than oral contraceptives. Insulin sensitivity was estimated from measurements during a 75-gram oral glucose tolerance test (oGTT) according to Matsuda and DeFronzo (ISIMats) [26]. This index mainly captures insulin effects in the liver and other peripheral organs (including skeletal muscle) [27]. We therefore used this index to capture peripheral insulin sensitivity (in contrast to brain/central insulin sensitivity). Body fat percentage was measured by Bioelectrical Impedance Analysis (BIA, single-frequency BIA device (50 kHz), manufacturer’s protocol: BIA 101 BIVA, Akern, Germany).

**Fig. 1:**
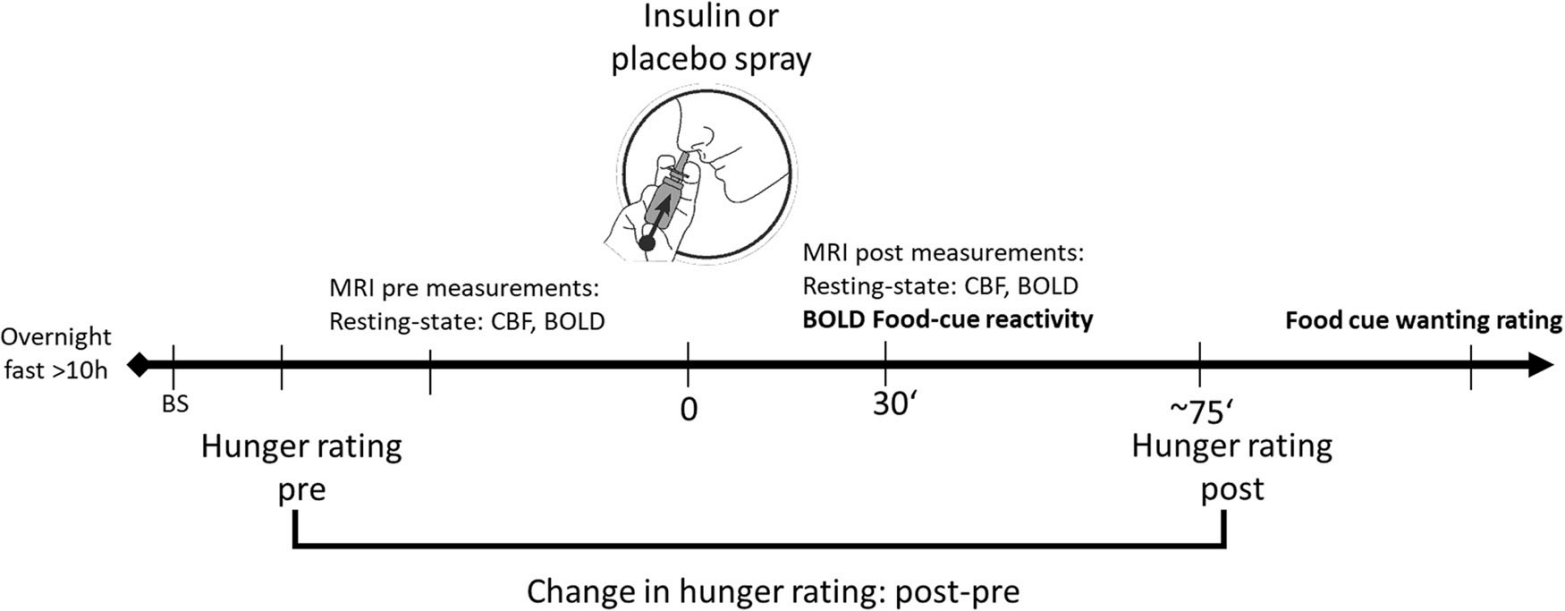
Scheme of test procedure. Cross-over design with intranasal insulin or placebo in a counter-balanced order. Hunger ratings were assessed at arrival and approximately 75 min after nasal spray application. The food cue viewing task during fMRI was followed by a task on a laptop to rate the food cues seen in the scanner based on wanting (i.e., desire to eat) and recognizability. Resting-state functional data sets were recorded at each visit before and 30 min after nasal spray application (results not reported here). BS blood sample, CBF cerebral blood flow, BOLD blood oxygenation-level dependent.

After the screening and oGTT measurement day, all subjects participated in two fMRI visits (Fig. 1) with a time-lag of 3–28 days. After an overnight fast of at least 10 h, visits were scheduled between 7 a.m. and 11 a.m. with intranasal insulin or intranasal placebo in a pseudo-randomized order. Insulin or placebo nasal spray application will be referred to as condition. After blood sampling, fMRI measurements were recorded under baseline (pre) and 30 min after nasal spray application (post).

A questionnaire addressing subjective feeling of hunger was assessed before and approximately 75 min after spray application using a visual analogue scale from 0 to 10 (0 = not hungry at all; 10 = very hungry). For the analysis, hunger ratings were baseline corrected, meaning the rating of the pre measurement was subtracted from the post measurement (Fig. 1).

At the end of each fMRI visit, participants rated the food cues, seen before in the scanner, in a wanting and a recognition task (described below).

To address trait eating behavior characteristics, the German Three Factor Eating Questionnaire (TFEQ), with the three subscales ‘Restraint eating/cognitive restraint of eating’, ‘disinhibition’ and ‘hunger’ [28], the eating disorder examination (EDE) [29] and the trait version of the Food Craving Questionnaire [30] was used (Supplementary Table 1).

#### Application intranasal insulin/ placebo

Participants received in total 160U of insulin (Insulin Actrapid; Novo Nordisk, Bagsvaerd, Denmark) or vehicle as placebo in a randomized fashion. The spray was administered over four minutes with two puffs per nostril every minute. Participants were blinded to the order of the conditions.

#### Imaging procedures

Scanning was conducted at a 3T whole-body Siemens scanner (Magnetom Prisma; Erlangen, Germany) with a 20-channel head coil. Neural food-cue reactivity using blood oxygen level dependent (BOLD)-fMRI was obtained after nasal spray application by using multi-band accelerated echo-planar imaging sequences, developed at the Center for Magnetic Resonance Research (CMRR) Minnesota, USA. The FCR consisted of two sessions, each lasting 5:30 min. Pictures were presented on a screen behind the scanner and were projected with a tilted mirror mounted on the head coil in the participant’s field of view. For fMRI measurements the following sequence parameters were used: TR = 1.5 s, TE = 34 ms, FOV = 192 mm², matrix 96 × 96, partial Fourier = 6/8, bandwidth = 2264 Hz/pixel, echo spacing = 0.55 ms, flip angle 70°, voxel size 2 × 2 × 2 mm^3^, slice thickness 2 mm, images were acquired in interleaved order with a multiband acceleration factor of 3. Each brain volume comprised 72 axial slices and each functional run contained 220 image volumes.

#### Food-cue task

An event-related design was used with high and low-caloric food cue pictures, presented in a pseudo-randomized order (software Presentation® (Version 10.2, www.neurobs.com)). Every picture was presented for 2 s with an interstimulus interval of 6–10 s. The pictures were separated by a grey screen with a black fixation circle or (every 6–7 pictures) a black fixation cross, in the middle of the screen. Participants were instructed to look at the pictures and immediately press a button when a cross appeared in between the pictures, to ensure attention and focus of the participants.

#### Stimulus material

A stimulus set of 60 food cues, 15 sweet high-caloric (e.g., donuts and cakes), 15 savory high-caloric (e.g., burger and pizza), 15 low-caloric sweet (e.g., fruit), and 15 savory low-caloric (e.g., vegetables and salads), was selected out of the freely available and standardized food cue database *food-pics* [31, 32] (*Supplementary Text and supplementary excel document*).

#### Recognition and wanting task

Approximately 15 min after the last fMRI measurement, participants performed outside of the scanner a computerized recognition (see *Supplementary Text*) and wanting task of the food cues seen during the fMRI measurement. The recognition task was used to control for attentiveness. For the wanting task (i.e., desire to eat), participants had to rate the food pictures by answering the question ‘how much they want to eat the food at that moment’, on a 5 point Likert scale going from ‘1-not at all’ to ‘5-very much’. Wanting ratings were calculated as sum of the wanting ratings (scale 1–5) for the 30 pictures per category (high and low-caloric). Wanting rating values are reported in Supplementary Table 2 and Supplementary Fig. 2a.

#### Image Processing of food-cue reactivity task

Pre-processing and statistical analysis of the fMRI data were performed using SPM12 (Wellcome Trust Centre for Neuroimaging, London, UK). Standard pre-processing including slice-timing, realignment, coregistration to the anatomical T1 weighted image, normalization into MNI space, and Gaussian spatial smoothing (FWHM: 6 mm) was done. A threshold of 2 mm maximum head motion displacement or 2° of any angular motion was applied. Finally, fMRI data were highpass (cut-off period 128 s) filtered and global AR(1) auto correlation correction was performed.

The FCR task was first analyzed according to the caloric content (event-related) of the food cues and secondly according to the individual participants’ wanting ratings.

#### Event-related analysis

For the calorie content, a design matrix was created individually for each participant for placebo and insulin day separately. For each condition, a separate regressor for low-caloric sweet, low-caloric savory, high-caloric sweet, and high-caloric savory was added in the model and convolved with a canonical hemodynamic response function and its time derivative. The movement regressors, separately for each session, were included as covariates in the model to account for possible movement-induced variance.

Individual contrast images were computed to estimate the activity changes for high-caloric pictures (sweet and savory together, 30 pictures in total) vs. low-caloric food cues (sweet and savory together, 30 pictures in total) (difference: high minus low-caloric food cue activity) on placebo and on insulin day. The individual contrasts for high minus low-caloric pictures were entered into a full factorial design for second level analysis.

#### Parametric modulation of the wanting ratings on food cue processing

For the analysis of the parametric modulation of the wanting ratings, a design matrix was created for each participant for placebo and insulin day separately. We used the individual wanting ratings for each food picture, independent of calorie content for parametric modulation of brain activity. Individual contrast images were computed according to the wanting ratings of the individual food cues. These contrast images were then entered into the full factorial models. We used the positive contrast, showing brain areas where the activity increased with increasing wanting ratings. (Three-dimensional representation in Supplementary Fig. 1).

### Statistical analyses

#### Food-cue task

We used two separate full factorial design models to investigate the effect of central insulin action on neural BOLD food-cue reactivity. The first model was based on the high minus low-caloric food contrasts; the second full factorial model was based on the wanting modulation contrasts. Both models included condition (insulin vs. placebo nasal spray) as a within-subject factor, BMI group (NW vs. OW) and sex group (female vs. male) as an in-between-subject factor, and age as a covariate.

A primary statistical threshold of *p* < 0.001 uncorrected and a *p* < 0.05 family wise error (FWE, based on Random Field Theory) corrected for multiple comparisons at a cluster level was applied. Additionally, small volume correction (SVC) was performed for regions recently identified as insulin sensitive [6], specifically the bilateral hypothalamus, the striatum, amygdala, hippocampus, insula and dorsolateral PFC. The masks were based on the *wfu* pick atlas (https://www.nitrc.org/projects/wfu_pickatlas/). For regions with SVC, reported *p*-values were adjusted by Bonferroni-correction for multiple comparisons (for the number of ROI’s).

For post hoc analyses and correlation analyses, differential responses were calculated by subtracting the individual regional brain activity of the placebo day from the insulin measurement. A *p* < 0.05 was considered significant after Bonferroni-Holm correction (Holm) for multiple testing.

#### Behavioral data

Data are given as mean ± SEM. For the analyses of the behavioral data and questionnaires, SPSS (IBM SPSS Statistics Version 26.0. Armonk, NY: IBM Corp) and R (Version 4.1.1R Core Team (2021). R: A language and environment for statistical computing. R Foundation for Statistical Computing, Vienna, Austria; URL https://www.R-project.org/.) were used.

Linear mixed-effect models including sex, BMI group, and condition as well as age (covariate), with subject as random intercept, were used to analyze hunger and wanting ratings. *F* and *p*-values were obtained by the lmerTest package [33] (Satterthwaite approximation for degrees of freedom), pairwise comparisons were performed by the emmeans package [34] with Bonferroni-Holm correction (Holm) for multiple testing. For post hoc analyses of group differences, differential (Insulin-Placebo) wanting and hunger ratings were used and *p*-values adjusted by Bonferroni–Holm correction.

If data were normally distributed, paired and two-sided *t*-tests were used. Otherwise, we used non-parametrical Kruskal Wallis H-Tests and Mann–Whitney U-Tests.

Furthermore, Spearman and Pearson correlations (depending if data were normally distributed or not) were performed to identify associations between central insulin action and peripheral insulin sensitivity and eating behavior characteristics adjusted for sex, BMI, and age (referred to as r_adj_ and p_adj_).

Mediation analysis of the relationship between peripheral insulin sensitivity, TFEQ-cognitive restraint and insular cortex activity was performed using PROCESS version 3.5 procedure in SPSS (www.afhayes.com). The significance of the mediation analysis (i.e., indirect effect *ab*) was estimated based on a bias-corrected bootstrap confidence interval (CI 95%, 5000 bootstrap samples).

For all analyses a *p* < 0.05 was considered significant.

## Results

### Central insulin effects on subjective feeling of hunger (VAS)

**Fig. 2:**
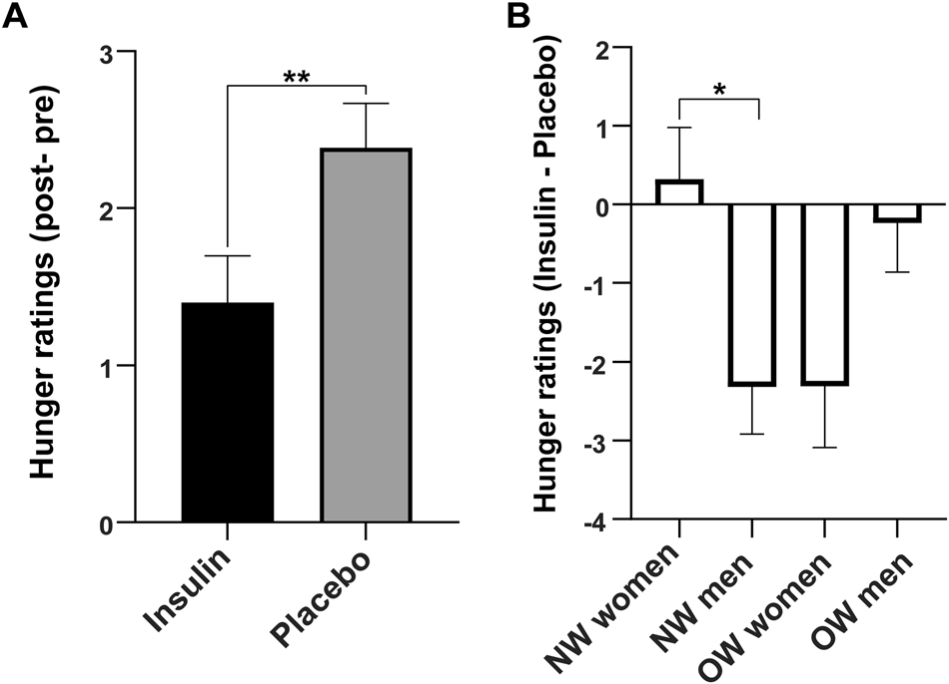
Significant reduction of hunger in response to intranasal insulin compared to placebo. **A** Bar plot shows change in hunger rating from before to after insulin or placebo spray application (post minus pre nasal spray) based on a visual analogue scale (in cm). **B** Bar plot shows change in hunger rating for insulin compared to placebo spray application (insulin day _post minus pre_ minus placebo day _post minus pre_). When comparing the four groups, NW men and OW women respond differently compared to NW women and OW men, even though, only the difference between NW women and NW men remained significant after correction for multiple comparisons. NW with normal weight, OW, with overweight and obesity; *p < 0.05 (Holm), **p < 0.01.

We observed a significant main effect of condition (F(1,56) = 10.712, *p* = 0.002) as well as a significant interaction between condition x sex x BMI group for the hunger ratings (F(1,56) = 11.494, *p* = 0.001). Post hoc paired *t*-tests (Supplementary Table 3a*)* revealed a significant reduction of the hunger ratings by intranasal insulin compared to placebo over all participants (T(56) = −3.273, *p* = 0.002; Fig. 2A), specifically in NW men (T(56) = −3.678, *p* = 0.002) and OW women (T(56) = −2.811, *p* = 0.020). Between group post hoc analyses showed that NW women and NW men differed significantly (T(55) = 3.034, *p* = 0.022; Fig. 2B, Supplementary Table 3b).

### Central insulin effects on wanting for high and low-caloric food cues

No main effect of condition was observed for high-caloric food wanting ratings (Supplementary Fig. 2b). However we observed a significant interaction between condition x sex (F(1,56) = 7.148, *p* = 0.01) and condition x sex x BMI (F(1,56) = 7.639, *p* = 0.008). Post hoc analyses showed that men displayed lower ratings than women (T(55) = −2.643, *p* = 0.01) and OW men lower wanting ratings for high-caloric food than OW women (T(55) = 3.357, *p* = 0.009; Supplementary Fig. 2c).

For low-caloric food, we observed higher wanting ratings in response to intranasal insulin compared to placebo (i.e., significant main effect of condition F(1,56) = 8.025, *p* = 0.006). No significant interaction effects were observed with condition (Supplementary Fig. 2d, e).

### Correlations with central insulin induced effects on wanting ratings

The differential (Insulin minus Placebo) wanting for high-caloric cues positively correlated with the percentage of body fat (*r* = 0.278, *p* = 0.033, r_adj._ = 0.295, p_adj._ = 0.027) and with TFEQ-cognitive restraint (*r* = 0.344, *p* = 0.008, r_adj._ = 0.283, p_adj._ = 0.036).

There were no significant correlations between wanting for low-caloric cues and percentage of body fat and cognitive restraint (*p* > 0.05).

### Central insulin action on neural BOLD activity based on high minus low-caloric food

There was a significant main effect of condition in the left amygdala (peak-voxel (MNI) x: −24, y: −8, z: −14); T(111) = 4.39, *p*_FWE-corr._ < 0.05, Supplementary Table 4 and Supplementary Fig. 3), with significantly higher BOLD activity after intranasal insulin compared to placebo.

**Fig. 3:**
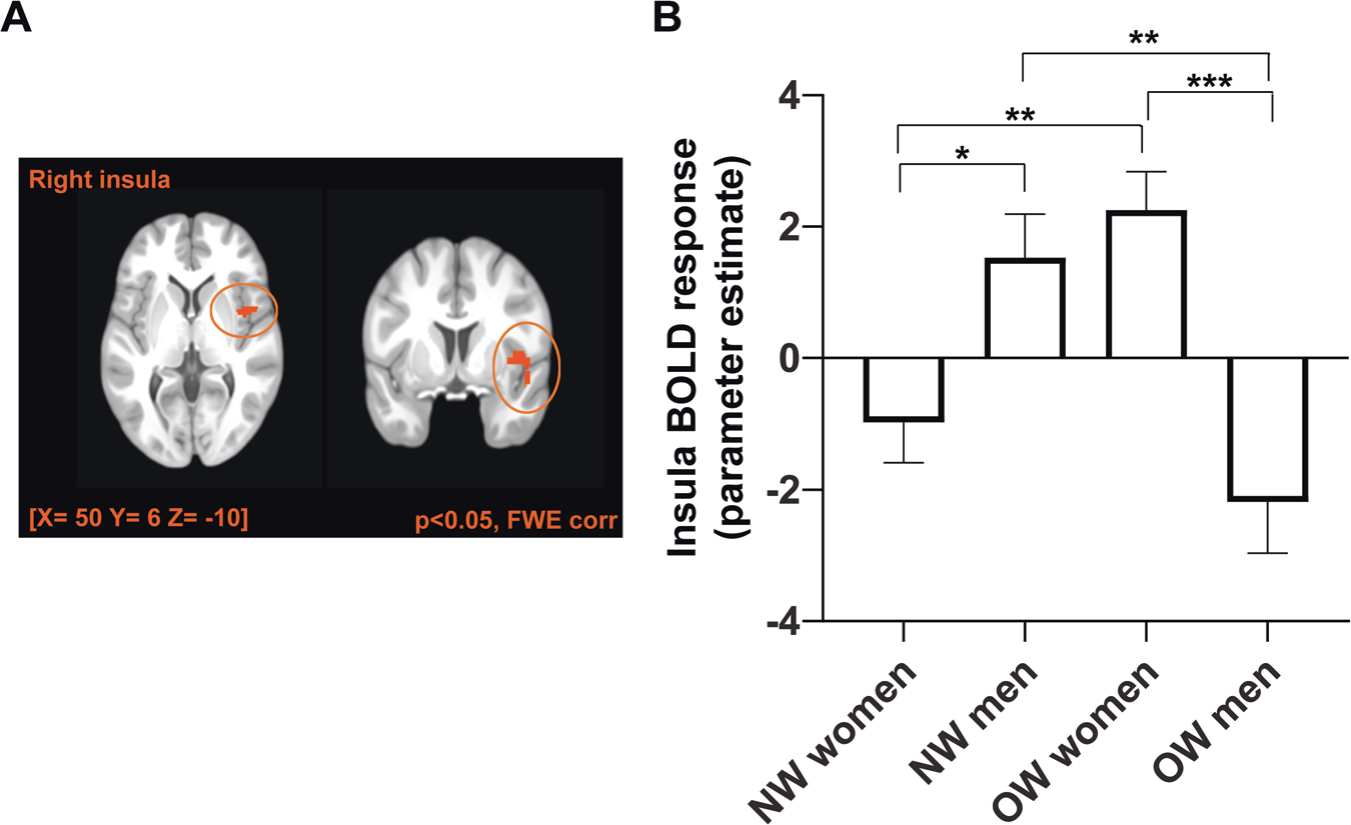
Central insulin action on BOLD response in the insular cortex. **A** Overlay shows a significant 3-way interaction between BMI x sex x condition (insulin versus placebo) in the right insular cortex in response to high minus low-caloric food cues (pFWE-corr. < 0.05). **B** Bar plot shows insular cortex BOLD activity (insulin minus placebo) for NW women, NW men, OW women and OW men separately. NW, with normal weight; OW, with overweight and obesity; *p < 0.5; **p < 0.01; ***p < 0.001 (Holm).

Significant interactions between BMI, sex and condition were found in the cerebellum/lingual gyrus (peak-voxel (MNI) [x: −14, y: −60, z: −12]; *T*-value = 4.66, *p*_FWE-corr._ < 0.05), precuneus (peak-voxel (MNI) [x: 8, y: −60, z: 60]; *T*-value = 4.51, *p*_FWE-corr._ < 0.05) and the insula (peak-voxel (MNI) [x: 50, y: 6, z: −10]; *T*-value = 4.71, *p*_FWE-corr._ < 0.05) (Supplementary Tables 4 & 5). Between group post hoc analyses showed that NW men and OW women displayed higher BOLD activations with intranasal insulin compared to placebo, whereas NW women and OW men showed lower activity in the insula and the precuneus (*p*_FWE-corr._ < 0.05; Supplementary Table 5b*)*. Food-cue reactivity in the insula is displayed in Fig. 3.

### Central insulin response in the insula correlates with behavioral and metabolic measures

The insula BOLD response correlated with peripheral insulin sensitivity (*r* = 0.293, *p* = 0.024 r_adj._ = 0.300, p_adj._ = 0.030) (Supplementary Fig. 4A). Hence, participants with higher peripheral insulin sensitivity showed an increased food-cue activation in the insular cortex in response to intranasal insulin.

Moreover, the insula response correlated positively with the TFEQ-cognitive restraint (*r* = 0.419, *p* = 0.001; r_adj._ = 0.466, p_adj._<0.001) *(*Supplementary Figs. 4B & 5*)* and the wanting ratings for the high-caloric cues (*r* = 0.257, *p* = 0.048; r_adj._ = 0.254, p_adj._ = 0.068). The correlation with the wanting ratings for high-caloric cues was driven by participants with overweight and obesity (NW: *r* = −0.025, *p* = 0.884, OW: *r* = 0.551, *p* = 0.006). No correlations were observed between behavioral measures and the central insulin response in the amygdala, cerebellum/lingual gyrus or precuneus.

**Fig. 4:**
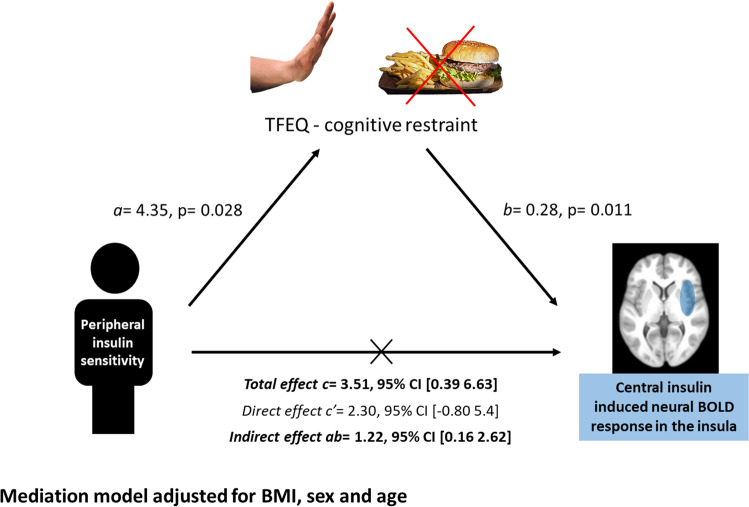
Cognitive restraint as mediator between peripheral and central insulin action. Graphic illustrates mediation model adjusted for sex, BMI and age. Cognitive restraint (based on three factor eating questionnaire) positively mediated the relationship between peripheral insulin sensitivity and the insular cortex BOLD activity (insulin minus placebo) in response to high-caloric food cues. Path coefficients and corresponding *p*-values are shown next to the arrows; path a indicates the relationship between peripheral insulin sensitivity and cognitive restraint, path b indicates the relationship between the cognitive restraint and the insula BOLD activity in response to high-caloric food cues; path ab indicates the indirect effect (not standardized) of peripheral insulin sensitivity on the insular cortex activity via the cognitive restraint score; path c’ indicate the direct effect of peripheral insulin sensitivity on the insular cortex activity.

Based on the correlations between the central insulin-induced BOLD response in the insula with peripheral insulin sensitivity and cognitive restraint, we tested, by mediation analyses, the process that underlies the observed relationships. We found a significant positive indirect effect (completely standardized indirect effect *ab* = 0.11, 95% CI [0.02 0.23]) of the TFEQ-cognitive restraint as a mediator between peripheral insulin sensitivity and differential insula BOLD activity. This indicates that cognitive restraint promotes the relationship between peripheral insulin sensitivity and central insulin action in the insular cortex (Fig. 4). Mediation models using the BOLD response in the insula or peripheral insulin sensitivity as a mediator did not indicate significant indirect effects. No direct effects were observed between peripheral insulin sensitivity and central insulin-induced BOLD response (see Fig. 4).

### Central insulin action on neural BOLD food-cue reactivity based on parametric modelling by individual wanting ratings

**Fig. 5:**
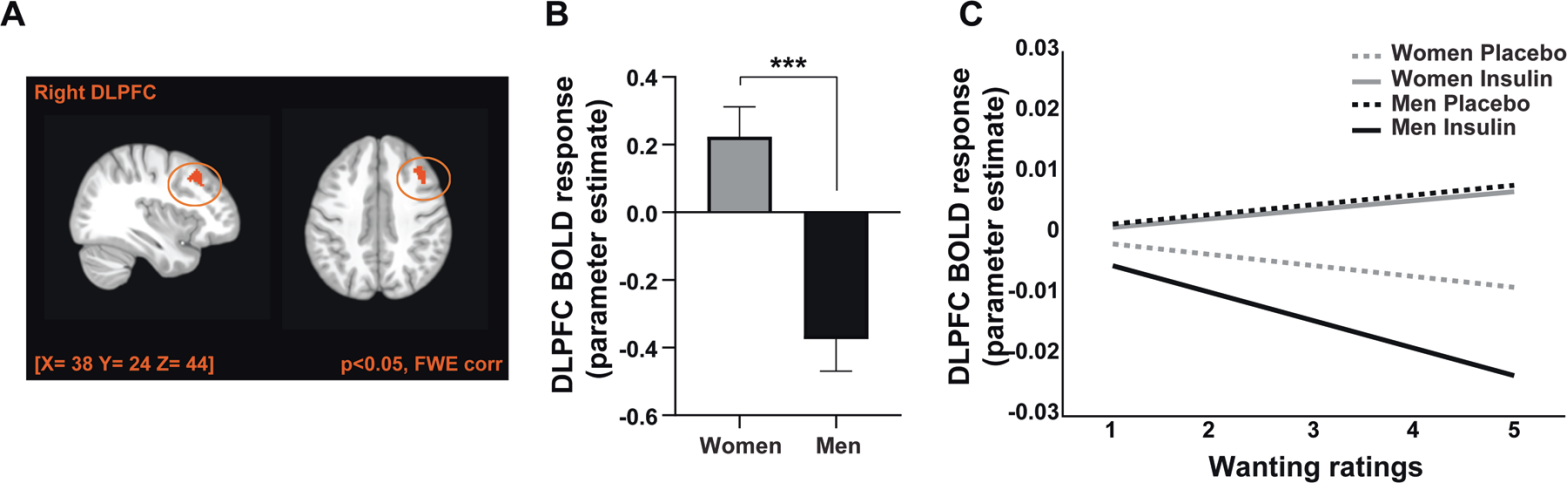
Insulin action on BOLD response in the dorsolateral prefrontal cortex (DLPFC). BOLD activity in the DLPFC is modulated by the individual wanting ratings for food cues. **A** Overlay shows significant 2-way interaction between sex and condition in the DLPFC with increasing wanting ratings for food cues (p_FWE-corr._ < 0.05). **B** Bar plot shows DLPFC BOLD response (insulin minus placebo) with increasing wanting ratings for women and men. Women showed significantly higher DLPFC BOLD activity than men. **C** Plot shows DLPFC BOLD activity for women and men with both insulin and placebo condition separately with increasing wanting ratings (1: low wanting; 5: high wanting, for visualization purposes only). ***p < 0.001.

No significant main effect of condition or interactions with BMI group were observed (*p* > 0.05) when we modelled brain responses according to the individual wanting ratings. We found a significant interaction between sex and condition in the dorsolateral frontal cortex (DLPFC) (right middle frontal gyrus, peak-voxel (MNI) [*x* = 38; *y* = 24; *z* = 44], *T*-value (peak) = 4.24, *p*_FWE-corr._ = 0.012, Fig. 5).

Within group comparisons revealed significant insulin vs. placebo effects in the DLPFC, for both women (T(29) = 2.542, *p* = 0.017) and men (T(29) = −3.968, *p* < 0.001). Between group post hoc comparisons revealed significant differences between women and men in the DLPFC response (T(58) = 4.634, *p* < 0.001). Men showed lower DLPFC activity in response to central insulin with increasing wanting ratings, while women showed higher DLPFC activity with increased wanting ratings.

The DLPFC response positively correlated with percent body fat (*r* = 0.444, *p* < 0.001; r_adj._ = −0.103, p_adj._ = 0.451) and with TFEQ-cognitive restraint (*r* = 0.373, *p* = 0.004; r_adj._ = 0.220, p_adj._ = 0.106).

## Discussion

In this study, we investigated the effect of central insulin action on neural BOLD food-cue reactivity in men and women with normal weight, overweight and obesity. Overall, central insulin action increased the BOLD response in the amygdala, while several other food-cue responsive regions [35], as the insular cortex, showed interactions between sex and obesity on how insulin affected FCR. The response in the DLPFC was modulated by individual wanting ratings of food cues. Here women showed higher BOLD activity than men in response to central insulin. On the behavioral level, we found that central insulin decreased the feeling of hunger and increased the desire to eat low-caloric food.

The amygdala has been reported as insulin-responsive in previous studies [36, 37] and we recently reported an increase in the amygdala in response to intranasal insulin in resting-state fMRI data [38]. Furthermore, the amygdala is implicated in taste and vision-related neural food reward pathways [39, 40], with higher activity in response to high-caloric food [41–43], particularly in the fasted state [35, 42]. Likewise, in the current study, central insulin led to a higher BOLD activity in the amygdala, in response to high versus low-caloric food cues. This implies that the amygdala responds to rewarding signals, which includes hormones as well as rewarding sensory signals from the environment.

Apart from the central insulin action in the amygdala, we found an interaction between sex and BMI on central insulin BOLD responsivity in several previous reported food-cue responsive cortical regions [35, 44–46]. Specifically, men with normal weight and women with overweight showed an increase in central insulin-induced BOLD response in the insula. Signals from the periphery and the environment converge in the insula to influence food intake [47]. Of note, several previous studies have identified either BMI or sex effects of central insulin action in the insular cortex. In young men with normal weight, central insulin induced an increase in regional blood flow [10], while men with overweight responded with a decrease [48]. Furthermore, in women with normal weight and obesity central insulin led to an increase in FCR in the insula [24]. Other studies, investigating food-cue reactivity, independent of insulin action, showed either BMI- or sex-related differences [41, 42, 49–51]. Two studies identified a similar food-cue response pattern in the insular cortex, with greater activation in the insula in participants with normal weight than in participants with overweight [49] and higher activity in the insula in women with overweight compared to normal weight [41]. This coincides with our study showing that women with overweight exemplify the greatest BOLD activity in the insular cortex in response to high-caloric cues, particularly in a fasted state.

Participants with high peripheral insulin sensitivity showed the highest central insulin-induced insular food-cue reactivity, which was fully mediated by cognitive restraint—a measure for the cognitive control of food intake. High scores in cognitive restraint correlate with a good maintenance of body weight or success in weight loss and lower BMI scores in people with overweight and obesity [52, 53]. Cognitive restraint may enhance the relationship between peripheral insulin sensitivity and central insulin BOLD response to food cues. Central insulin was shown to influence dopamine signaling and reduce hedonic aspects of food [9, 11, 12]. Thus, cognitive restraint could further affect the subjective value and rewarding effect of food.

Based on our findings, we postulate that the insula BOLD response in men with normal weight was primarily driven by physiological signals (i.e., central insulin), while the response of women with overweight was additionally driven by environmental cues and cognitive processes [54]. Noteworthy, women with normal weight did not show an increase in insula BOLD activity with intranasal insulin. This could be due to the fact that we performed our study in the fasted state. Studies in the postprandial state identified a central insulin induced reduction in appetite ratings [23, 24] and increased insula activity [24] in women of normal and overweight. Hence, the nutritional state could additionally modulate the brain’s response to food cues [35, 42, 51, 55–57], though, the detailed underlying mechanisms remain unclear. Furthermore, hormonal fluctuations during the menstrual cycle have shown to influence peripheral and central insulin sensitivity [58, 59]. Hence, further studies are needed to evaluate the complex interplay of sex hormones and nutritional state on the brain response to physiological and environmental cues.

On a merely behavioral level, central insulin action led to the strongest decrease in the hunger ratings in men with normal weight and women with overweight. Accordingly, previous studies, mostly in men with normal weight, described decreased ratings for appetite or hunger and food intake following intranasal insulin [8, 20]. Surprisingly, in the current study, we identified a general increase in low-caloric wanting ratings with intranasal insulin. This expands previous findings, showing that central insulin not only decreases hunger [8, 20] and food wanting for high-caloric food [8, 11, 60] but can also enhance wanting for low-caloric food. This could further corroborate that central insulin is a rewarding signal [54].

Furthermore, central insulin action led to a significant sex-dependent DLPFC BOLD response with increasing desire (i.e., wanting) for food cues. Women showed an increase in activity with increasing wanting following intranasal insulin, while men showed a decrease. The prefrontal cortex plays a crucial role in decision-making and cognitive control of food intake [61, 62] and is highly responsive to hormonal signals like insulin [8, 25]. The sex differences of central insulin action in the DLPFC further support the notion that women and men differ in central insulin signaling when eating behavior-related cognitive processes are involved [6, 20]. Meaning that in men, central insulin action reduces prefrontal activity for high wanted food cues and decreases hunger. This could lead to a decrease of food intake, as described in men [21] and male rats [63, 64]. Whereas in women, central insulin action seems to be influenced by cognitive processes related food choice, which is further supported by the positive association between DLPFC BOLD activity and cognitive restraint. Hence, physiological signals as central insulin regulate homeostasis and appetite in men, while in women there might be a dissociation between physiological and cognitive signals.

## Conclusion

Obesity and sex seem to play a major role in central insulin-mediated neural BOLD food-cue reactivity. Our study shows a complex interaction between sex and obesity during neural FCR, which is associated with peripheral insulin sensitivity and cognitive restraint, which indicates that further factors likely contribute. Furthermore, neural activity modulated by the desire for food cues revealed pronounced sex differences in prefrontal activity. This further supports the hypothesis that insulin signaling in the brain differs between women and men, especially in the regulation of cognitive and hedonic processes.

### Limitations

In our current study, we could not analyze the impact of menstrual cycle or contraceptive medication as the sample size was not large enough for further stratified analyses. Nonetheless, it is known that peripheral insulin sensitivity changes throughout the cycle [58] and also eating behavior and preferences may change [18] and should therefore be addressed in further experiments. Furthermore, peripheral insulin sensitivity was not assessed through hyperinsulinemic-euglycemic clamp but estimated with the widely-used Matsuda index from repeated insulin and glucose measurements during an oGTT [65].

## Supplementary information

Supplements (pdf)

Food cue characteristics (Supplementary excel table)

## Data Availability

The data are not publicly available due to them containing information that could compromise research participant privacy/consent. The authors will share them by request from any qualified investigator after the completion of a data-sharing agreement.

## References

[1] Baskin DG, Figlewicz DP, Woods SC, Porte D, Dorsa DM (1987). Insulin in the brain. Annu Rev Physiol.

[2] Schwartz MW, Figlewicz DP, Baskin DG, Woods SC, Porte D (1992). Insulin in the brain: a hormonal regulator of energy balance. Endocr Rev.

[3] Bruning JC, Gautam D, Burks DJ, Gillette J, Schubert M, Orban PC (2000). Role of brain insulin receptor in control of body weight and reproduction. Science.

[4] Kleinridders A, Pothos EN (2019). Impact of brain insulin signaling on dopamine function, food intake, reward, and emotional behavior. Curr Nutr Rep.

[5] Hallschmid M (2021). Intranasal insulin. J Neuroendocrinol.

[6] Kullmann S, Kleinridders A, Small DM, Fritsche A, Haring HU, Preissl H (2020). Central nervous pathways of insulin action in the control of metabolism and food intake. Lancet Diabetes Endocrinol.

[7] Heni M, Wagner R, Kullmann S, Gancheva S, Roden M, Peter A (2017). Hypothalamic and striatal insulin action suppresses endogenous glucose production and may stimulate glucose uptake during hyperinsulinemia in lean but not in overweight men. Diabetes..

[8] Kullmann S, Heni M, Veit R, Scheffler K, Machann J, Haring HU (2015). Selective insulin resistance in homeostatic and cognitive control brain areas in overweight and obese adults. Diabetes Care.

[9] Kullmann S, Blum D, Jaghutriz BA, Gassenmaier C, Bender B, Haring HU (2021). Central insulin modulates dopamine signaling in the human striatum. J Clin Endocrinol Metab.

[10] Schilling TM, Ferreira de Sa DS, Westerhausen R, Strelzyk F, Larra MF, Hallschmid M (2014). Intranasal insulin increases regional cerebral blood flow in the insular cortex in men independently of cortisol manipulation. Hum Brain Mapp.

[11] Tiedemann LJ, Schmid SM, Hettel J, Giesen K, Francke P, Buchel C (2017). Central insulin modulates food valuation via mesolimbic pathways. Nat Commun.

[12] Edwin Thanarajah S, Iglesias S, Kuzmanovic B, Rigoux L, Stephan KE, Bruning JC (2019). Modulation of midbrain neurocircuitry by intranasal insulin. Neuroimage..

[13] Zhang H, Hao Y, Manor B, Novak P, Milberg W, Zhang J (2015). Intranasal insulin enhanced resting-state functional connectivity of hippocampal regions in type 2 diabetes. Diabetes..

[14] Berthoud HR (2007). Interactions between the “cognitive” and “metabolic” brain in the control of food intake. Physiol Behav.

[15] Belfort-DeAguiar R, Seo D (2018). Food cues and obesity: overpowering hormones and energy balance regulation. Curr Obes Rep.

[16] Hermann P, Gal V, Kobor I, Kirwan CB, Kovacs P, Kitka T (2019). Efficacy of weight loss intervention can be predicted based on early alterations of fMRI food cue reactivity in the striatum. Neuroimage Clin.

[17] Liu S, Borgland SL (2019). Insulin actions in the mesolimbic dopamine system. Exp Neurol.

[18] Chao AM, Loughead J, Bakizada ZM, Hopkins CM, Geliebter A, Gur RC (2017). Sex/gender differences in neural correlates of food stimuli: a systematic review of functional neuroimaging studies. Obes Rev.

[19] Killgore WD, Weber M, Schwab ZJ, Kipman M, DelDonno SR, Webb CA (2013). Cortico-limbic responsiveness to high-calorie food images predicts weight status among women. Int J Obes.

[20] Benedict C, Kern W, Schultes B, Born J, Hallschmid M (2008). Differential sensitivity of men and women to anorexigenic and memory-improving effects of intranasal insulin. J Clin Endocrinol Metab.

[21] Jauch-Chara K, Friedrich A, Rezmer M, Melchert UH, H GS-E, Hallschmid M (2012). Intranasal insulin suppresses food intake via enhancement of brain energy levels in humans. Diabetes.

[22] Hallschmid M, Benedict C, Schultes B, Fehm HL, Born J, Kern W (2004). Intranasal insulin reduces body fat in men but not in women. Diabetes..

[23] Hallschmid M, Higgs S, Thienel M, Ott V, Lehnert H (2012). Postprandial administration of intranasal insulin intensifies satiety and reduces intake of palatable snacks in women. Diabetes..

[24] Schneider E, Spetter MS, Martin E, Sapey E, Yip KP, Manolopoulos KN, et al. The effect of intranasal insulin on appetite and mood in women with and without obesity: an experimental medicine study. Int J Obes. 2022. [Online ahead of print]. 10.1038/s41366-022-01115-1 PMC9239904 35397638

[25] Kullmann S, Frank S, Heni M, Ketterer C, Veit R, Haring HU (2013). Intranasal insulin modulates intrinsic reward and prefrontal circuitry of the human brain in lean women. Neuroendocrinology..

[26] Matsuda M, DeFronzo RA (1999). Insulin sensitivity indices obtained from oral glucose tolerance testing: comparison with the euglycemic insulin clamp. Diabetes Care.

[27] Gutch M, Kumar S, Razi SM, Gupta KK, Gupta A (2015). Assessment of insulin sensitivity/resistance. Indian J Endocrinol Metab.

[28] Pudel V, Westenhöfer J, Fragebogen zum Eßverhalten (FEV)-Handanweisung: Göttingen; Verlag für Psychologie Dr. CJ Hogrefe; 1989.

[29] Hilbert A, Tuschen-Caffier B, Karwautz A, Niederhofer H, Munsch S (2007). Eating disorder examination-questionnaire: Psychometric properties of the German version. Diagnostica..

[30] Nijs IM, Franken IH, Muris P (2007). The modified Trait and State Food-Cravings Questionnaires: development and validation of a general index of food craving. Appetite..

[31] Blechert J, Meule A, Busch NA, Ohla K (2014). Food-pics: an image database for experimental research on eating and appetite. Front Psychol.

[32] Blechert J, Lender A, Polk S, Busch NA, Ohla K (2019). Food-Pics_Extended-An image database for experimental research on eating and appetite: additional images, normative ratings and an updated review. Front Psychol.

[33] Kuznetsova A, Brockhoff PB, Christensen RHB (2017). lmerTest package: tests in linear mixed effects models. Journal of Statistical Software.

[34] Lenth RV emmeans: Estimated Marginal Means, aka Least-Squares Means. 2022. R package version 1.7.2. https://CRAN.R-project.org/package=emmeans.

[35] van der Laan LN, de Ridder DT, Viergever MA, Smeets PA (2011). The first taste is always with the eyes: a meta-analysis on the neural correlates of processing visual food cues. Neuroimage..

[36] Areias MF, Prada PO (2015). Mechanisms of insulin resistance in the amygdala: influences on food intake. Behav Brain Res.

[37] Soto M, Cai W, Konishi M, Kahn CR (2019). Insulin signaling in the hippocampus and amygdala regulates metabolism and neurobehavior. Proc Natl Acad Sci USA.

[38] Kullmann S, Veit R, Peter A, Pohmann R, Scheffler K, Haring HU (2018). Dose-dependent effects of intranasal insulin on resting-state brain activity. J Clin Endocrinol Metab.

[39] Avery JA, Liu AG, Ingeholm JE, Riddell CD, Gotts SJ, Martin A (2020). Taste quality representation in the human brain. J Neurosci.

[40] Rolls ET, The orbitofrontal cortex, food reward, body weight, and obesity. Soc Cogn Affect Neurosci. 2021;nsab044. [Online ahead of print]. 10.1093/scan/nsab044 PMC9997078 33830272

[41] Stoeckel LE, Weller RE, Cook EW, Twieg DB, Knowlton RC, Cox JE (2008). Widespread reward-system activation in obese women in response to pictures of high-calorie foods. Neuroimage..

[42] Goldstone AP, Prechtl de Hernandez CG, Beaver JD, Muhammed K, Croese C, Bell G (2009). Fasting biases brain reward systems towards high-calorie foods. Eur J Neurosci.

[43] Fletcher PC, Napolitano A, Skeggs A, Miller SR, Delafont B, Cambridge VC (2010). Distinct modulatory effects of satiety and sibutramine on brain responses to food images in humans: a double dissociation across hypothalamus, amygdala, and ventral striatum. J Neurosci.

[44] Tang DW, Fellows LK, Small DM, Dagher A (2012). Food and drug cues activate similar brain regions: a meta-analysis of functional MRI studies. Physiol Behav.

[45] Drummen M, Dorenbos E, Vreugdenhil ACE, Raben A, Westerterp-Plantenga MS, Adam TC (2019). Insulin resistance, weight, and behavioral variables as determinants of brain reactivity to food cues: a Prevention of Diabetes through Lifestyle Intervention and Population Studies in Europe and around the World - a PREVIEW study. Am J Clin Nutr.

[46] Morys F, Garcia-Garcia I, Dagher A, Is obesity related to enhanced neural reactivity to visual food cues? A review and meta-analysis. Soc Cogn Affect Neurosci. 2020;nsaa113. [Online ahead of print]. 10.1093/scan/nsaa113 PMC9997070 32785578

[47] de Araujo IE, Schatzker M, Small DM (2020). Rethinking food reward. Annu Rev Psychol.

[48] Wingrove JO, O’Daly O, Forbes B, Swedrowska M, Amiel SA, Zelaya FO (2021). Intranasal insulin administration decreases cerebral blood flow in cortico-limbic regions: A neuropharmacological imaging study in normal and overweight males. Diabetes Obes Metab.

[49] Dimitropoulos A, Tkach J, Ho A, Kennedy J (2012). Greater corticolimbic activation to high-calorie food cues after eating in obese vs. normal-weight adults. Appetite..

[50] Rothemund Y, Preuschhof C, Bohner G, Bauknecht HC, Klingebiel R, Flor H (2007). Differential activation of the dorsal striatum by high-calorie visual food stimuli in obese individuals. Neuroimage..

[51] Frank S, Laharnar N, Kullmann S, Veit R, Canova C, Hegner YL (2010). Processing of food pictures: influence of hunger, gender and calorie content. Brain Res.

[52] Loffler A, Luck T, Then FS, Luppa M, Sikorski C, Kovacs P (2015). Age- and gender-specific norms for the German version of the Three-Factor Eating-Questionnaire (TFEQ). Appetite..

[53] Johnson F, Pratt M, Wardle J (2012). Dietary restraint and self-regulation in eating behavior. Int J Obes.

[54] Stouffer MA, Woods CA, Patel JC, Lee CR, Witkovsky P, Bao L (2015). Insulin enhances striatal dopamine release by activating cholinergic interneurons and thereby signals reward. Nat Commun.

[55] Geliebter A, Pantazatos SP, McOuatt H, Puma L, Gibson CD, Atalayer D (2013). Sex-based fMRI differences in obese humans in response to high vs. low energy food cues. Behav Brain Res.

[56] Liu S, Borgland SL (2015). Regulation of the mesolimbic dopamine circuit by feeding peptides. Neuroscience..

[57] Smeets PAM, Dagher A, Hare TA, Kullmann S, van der Laan LN, Poldrack RA (2019). Good practice in food-related neuroimaging. Am J Clin Nutr.

[58] MacGregor KA, Gallagher IJ, Moran CN (2021). Relationship Between Insulin Sensitivity and Menstrual Cycle Is Modified by BMI, Fitness, and Physical Activity in NHANES. J Clin Endocrinol Metab.

[59] Benkendorff CFC, Hummel J, Vosseler A, Kullmann S, Fritsche L, Birkenfeld AL (2020). 150-OR: Brain Insulin Sensitivity Is Modulated by Menstrual Cycle. Diabetes..

[60] de Sá DSF, Schulz A, Streit FE, Turner JD, Oitzl MS, Blumenthal TD (2014). Cortisol, but not intranasal insulin, affects the central processing of visual food cues. Psychoneuroendocrinology..

[61] Gluck ME, Viswanath P, Stinson EJ (2017). Obesity, appetite, and the prefrontal cortex. Curr Obes Rep.

[62] Krawczyk DC (2002). Contributions of the prefrontal cortex to the neural basis of human decision making. Neurosci Biobehav Rev.

[63] Air EL, Benoit SC, Blake Smith KA, Clegg DJ, Woods SC (2002). Acute third ventricular administration of insulin decreases food intake in two paradigms. Pharmacol Biochem Behav.

[64] Clegg DJ, Riedy CA, Smith KA, Benoit SC, Woods SC (2003). Differential sensitivity to central leptin and insulin in male and female rats. Diabetes..

[65] Hudak S, Huber P, Lamprinou A, Fritsche L, Stefan N, Peter A (2021). Reproducibility and discrimination of different indices of insulin sensitivity and insulin secretion. PLoS One.

