# Supplementary material for: Sex differences in central insulin action: Effect of intranasal insulin on neural food cue reactivity in adults with normal weight and overweight
Source: Int J Obes (Lond). 2022 Jun 17;46(9):1662–70. doi: 10.1038/s41366-022-01167-3 (PMC9395264; doi:10.1038/s41366-022-01167-3)
Supplement: Supplementary file 1 — Supplements (pdf) [file 41366_2022_1167_MOESM1_ESM.pdf]

Supplements: Sex differences in central insulin action: Effect of intranasal insulin on neural food cue reactivity in adults with normal weight and overweight

Lore Wagner[1, 2 *], *Ralf Veit*[1,2], *Louise Fritsche*[1,2], *Hans-Ulrich Häring*[1,2,3], *Andreas Fritsche*[1,2,3,4], *Andreas L. Birkenfeld*[1,2,3], *Martin Heni*[1,2,3,5,6], *Hubert Preissl*[1,2,3,7], *Stephanie Kullmann*[1,2,3]

[1]Institute for Diabetes Research and Metabolic Diseases of the Helmholtz Center Munich at the University of Tübingen, Tübingen, Germany
[2]German Center for Diabetes Research (DZD e.V.), Tübingen, Germany
[3]Department of Internal Medicine, Division of Endocrinology, Diabetology and Nephrology, Eberhard Karls University Tübingen, Tübingen, Germany
[4]Nutritional and Preventive Medicine, Eberhard Karls University Tübingen, Tübingen, Germany
[5]Institute for Clinical Chemistry and Pathobiochemistry, University Hospital Tübingen, Tübingen, Germany
[6]Department of Internal Medicine I, Division of Endocrinology and Diabetology, Ulm University Hospital, Ulm, Germany
[7]Institute for Diabetes and Obesity, Helmholtz Diabetes Center, Helmholtz Center Munich, German Research Center for Environmental Health (GmbH), Neuherberg, Germany

---

* Corresponding author at: Institute for Diabetes Research and Metabolic Diseases of the Helmholtz Center Munich at the University of Tübingen, Otfried Müller Str. 47, 72076 Tübingen, Germany.
Email: Lore.wagner@uni-tuebingen.de

Supplements Methods

Supplementary Text

Stimulus Material

High and low-caloric pictures were matched for RGB-color distribution, intensity, contrast, complexity and object size. Concerning the nutritional values, the high-caloric cues had significantly higher amounts of protein, fat, carbs and kcal, as well per 100 g of the presented food as in total. The amount of presented food in the image (measured in grams/total) did not differ between high and low-caloric cues. (*please see excel sheet*)

Recognition task

Participants performed a recognition task of the food cues seen during the fMRI measurement on a laptop outside of the scanner. First, participants were shown 100 pictures (60 defined as old, as they were recently seen in scanner, and 40 new ones) for which they had to indicate on a 5 point Likert-scale ('1 – the picture is new for sure', '3- I do not know', '5- the picture is old for sure'), if they had seen them during the task in the scanner. The recognition task was used to control for the attentiveness of the participants and not further analyzed. The order of the pictures was randomized.

Parametric modulation of the wanting ratings on food cue processing

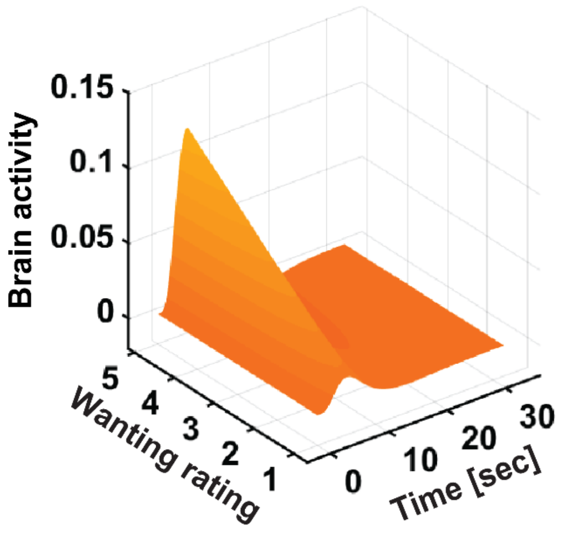

Supplementary Figure 1*: The individual wanting ratings for each food picture, independent of calorie content were used for parametric modulation of brain activity. We used the positive contrast, showing brain areas where the activity increased with increasing wanting ratings. Three-dimensional representation of parametric correlation of wanting (for the 60 food cues) with signal changes (example of one participant, for visualization purposes only) are shown in the figure.*

Supplements Results

Behavioral Results

Sex and BMI effects on Trait-Questionnaires of eating behavior characteristics

Supplementary Table 1: Trait- Questionnaires of eating behavior characteristics

	**Normal weight (NW)**		**Overweight/obesity (OW)**		
	**women**	**men**	**women**	**men**	**p-value**
**Eating disorder examination (EDE) questionnaire**					
**- Restraint**	3.6 *(0.73)*	2.63 *(1.12)*	9 *(2.36)*	2.54 *(1.16)*	**0.014**
**- Eating concern**	1.63 *(0.55)*	5.31 *(2.7)*	4.78 *(1.95)*	2.15 *(1.01)*	0.586
**- Weight concern**	1.6 *(0.55)*	0.25 *(0.11)*	5.9 *(2.17)*	1.38 *(0.87)*	**0.002**
**- Shape concern**	3.95 *(0.74)*	2.38 *(0.78)*	9.2 *(2.14)*	4.08 *(1.87)*	**0.028**
**German three factor eating questionnaire (TFEQ)**					
**- Cognitive restraint**	6.95 *(0.82)*	6.18 *(0.71)*	11.5 *(0.93)*	5.33 *(1.05)*	**0.002**
**- Disinhibition**	4.68 *(0.58)*	3.88 *(0.48)*	5.8 *(0.96)*	5.17 *(0.89)*	0.415
**- Hunger**	4.16 *(0.70)*	3.82 *(0.58)*	4.9 *(1.09)*	4.09 *(0.90)*	0.929
**Food craving questionnaire trait (FCQ-T)**					
**-Total**	80.89 *(5.30)*	76 *(4.22)*	85.1 *(10.14)*	64.92 *(10.31)*	0.132

Values in the Table given as mean (SEM).
p-values: non-parametrical Kruskal-Wallis-H-Test, uncorrected for multiple testing

For the EDE-weight concern, we found significant BMI x sex group differences ($\chi2(3)= 14.42$, $p= 0.002$). Post hoc tests showed that women with overweight and obesity had significantly higher scores than all three other groups ($p< 0.05$) and that women with normal weight had significantly higher scores than men with normal weight ($p= 0.031$). In the EDE, in the subscales 'Restraint' and 'Shape concern' as well as in the TFEQ- Cognitive restraint, women with overweight and obesity rated significantly higher than the three other groups (EDE-restraint: $\chi2(3)= 10.67$, $p= 0.014$, EDE-Shape concern: $\chi2(3)= 9.13$, $p= 0.028$, TFEQ-cognitive restraint: $\chi2(3)= 15.31$, $p= 0.002$). There was no significant difference between the three other groups. No significant differences were observed between groups in total food craving (FCQ-T).

Central insulin effect on hunger ratings and wanting ratings of high and low-caloric food cues

Supplementary Table 2: Wanting and hunger ratings in the four BMI and sex groups (Mean (± SEM))

Note: For accuracy reasons, wanting ratings are shown as sum of the wanting ratings (scale 1-5) for the 30 pictures per category (high/low-caloric). If of interest, values can be divided by 30 (number of pictures per category) to obtain a mean wanting rating value.

			**Normal weight (NW)**		**Overweight/obesity (OW)**	
			**women**	**men**	**women**	**men**
**Wanting ratings**						
	Low-caloric	Placebo	100.85 *(5.82)*	94.06 *(5.13)*	123.1 *(6.76)*	87.23 *(4.91)*
		Insulin	108.7 *(5.19)*	99.53 *(4.3)*	124.8 *(8.12)*	88.92 *(5.86)*
		Delta	7.85 *(2.94)*	5.47 *(2.79)*	1.7 *(2.67)*	1.69 *(2.23)*
	High-caloric	Placebo	78.6 *(5.83)*	87.76 *(5.45)*	76.4 *(8.32)*	88.46 *(6.35)*
		Insulin	82.8 *(5.99)*	92.29 *(5.73)*	87.3 *(8.75)*	79.54 *(6.13)*
		Delta	4.2 *(3.04)*	4.53 *(2.92)*	10.9 *(4.06)*	-8.92 *(4.47)*
**Hunger ratings [cm]**	Placebo	Pre	2.67 *(0.54)*	2.9 *(0.6)*	3.07 *(0.72)*	3.66 *(0.72)*
		Post	4.83 *(0.58)*	5.41 *(0.65)*	6.62 *(0.87)*	5.32 *(0.73)*
		Change	2.17 *(0.53)*	2.51 *(0.37)*	3.55 *(0.62)*	1.65 *(0.73)*
	Insulin	Pre	3.36 *(0.52)*	4.66 *(0.67)*	4.08 *(0.88)*	2.94 *(0.58)*
		Post	5.85 *(0.53)*	4.86 *(0.66)*	5.32 *(0.9)*	4.35 *(0.71)*
		Change	2.49 *(0.6)*	0.19 *(0.45)*	1.24 *(0.49)*	1.42 *(0.58)*

Central insulin effects on wanting ratings of high and low-caloric food cues

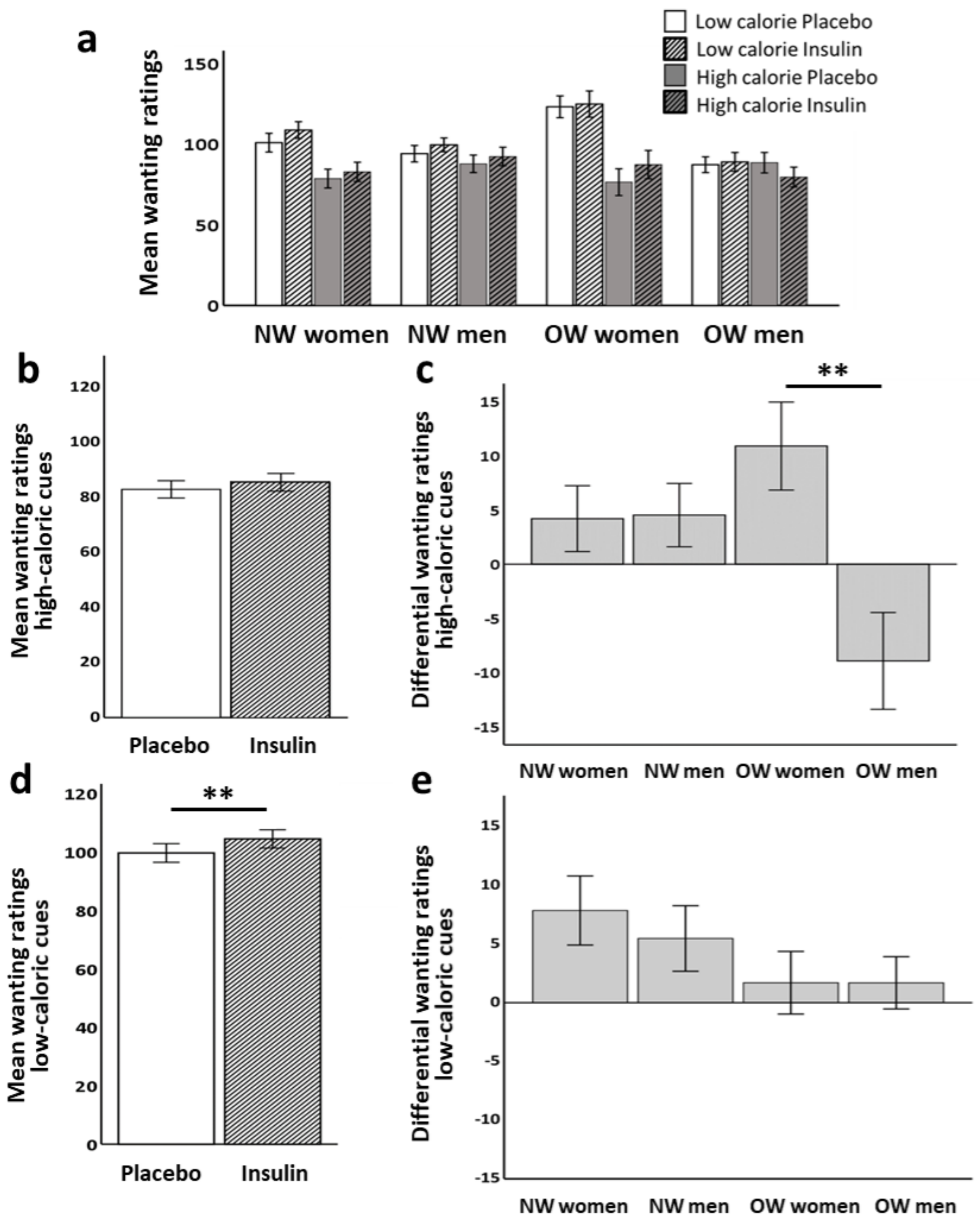

***Supplementary Figure 2**: Wanting ratings for high and low-caloric food cues after intranasal insulin and placebo application. (a) For display only, bar plot shows mean sum of the wanting ratings (± SEM) in the four BMI and sex groups (b) Over all participants, bar plot shows mean wanting rating for high-caloric cues; no significant differences were observed after insulin compared to placebo application over all participants (p>0.05); however, significant interactions were observed between condition (insulin versus placebo), sex and BMI (p<0.05, see main document). For post hoc analyses, differential wanting ratings were used (insulin minus placebo). (c) Bar plot shows differential wanting ratings for high-caloric cues in the four*

*BMI and sex groups. Men with overweight and obesity revealed lower differential wanting ratings for high-caloric cues compared to all other groups, but after Holm-correction for multiple testing, only OW women and OW men differed significantly. (OW women > OW men: T(55)= 3.357, p= 0.009; NW men vs. OW men: T(55)= 2.482, p= 0.06; NW women vs. OW men: T(55)=2.573, p= 0.06, holm-correction for 6 tests). (d) Over all participants, bar plot displays mean wanting ratings for low-caloric food cues showing higher wanting ratings in response to intranasal insulin compared to placebo (T(56)= 2.833, p= 0.006). (e) Bar plot shows differential wanting ratings for low-caloric cues in the four BMI and sex groups. No significant interaction effects were found with condition. Abbreviations: NW, with normal weight; OW, with overweight and obesity. ** p < 0.01, error bars: ± SEM*

Central insulin effects on subjective feeling of hunger (VAS) based on significant 3-way interaction (BMI x sex x condition, adjusted for age)

Supplementary Table 3a: Within group post hoc comparisons: Main effect of condition (insulin versus placebo)

	NW women		NW men		OW women		OW men	
	T(56)	p-value	T(56)	p-value	T(56)	p-value	T(56)	p-value
**Hunger ratings (VAS)**	0.551	1	-3.678	**0.002**	-2.811	**0.020**	-0.331	1

Degrees-of-freedom method: Satterthwaite, p-value adjustment: Bonferroni-Holm method for 4 tests

Supplementary Table 3b: Between group post hoc comparisons of differential hunger ratings. i.e. insulin minus placebo (post-pre)

	NW women vs. NW men		NW women vs. OW women		NW women vs. OW men		NW men vs. OW women		NW men vs. OW men		OW women vs. OW men	
	T(55)	p-value	T(55)	p-value	T(55)	p-value	T(55)	p-value	T(55)	p-value	T(55)	p-value
**Hunger ratings**	3.034	**0.022**	2.592	0.06	0.616	1	-0.004	1	-2.096	0.163	-1.855	0.207

Degrees-of-freedom method: Satterthwaite, p-value adjustment: Boferroni-Holm method for 6 tests

Imaging Results

Supplementary Table 4: Neural food cue BOLD reactivity in response to intranasal insulin compared to placebo for high- minus low-caloric food contrast

Regions	Hemisphere	T-value (peak)	P[*]	MNI-coordinates [mm] [a]		
				x	y	z
**Insulin > Placebo**						
Amygdala	L	4.39	0.012[b]	-24	-8	-14
**Interaction BMI x sex x condition**						
Insula	R	4.71	0.013	50	6	-10
Cerebellum/ Lingual	L	4.66	0.008	-14	-60	-12
Precuneus	R	4.51	<0.001	8	-60	60

** p < 0.05, FWE-corrected for multiple comparison (whole-brain); [a] Montreal Neurological Institute (peak-voxel); [b] Small volume corrected (mask) with Bonferroni correction for the number of ROI's: specifically the bilateral hypothalamus, the striatum, amygdala, hippocampus, insula and dorsolateral PFC. The masks were based on the wfu pick atlas (https://www.nitrc.org/projects/wfu_pickatlas/).*

Higher amygdalar food-cue reactivity in response to central insulin

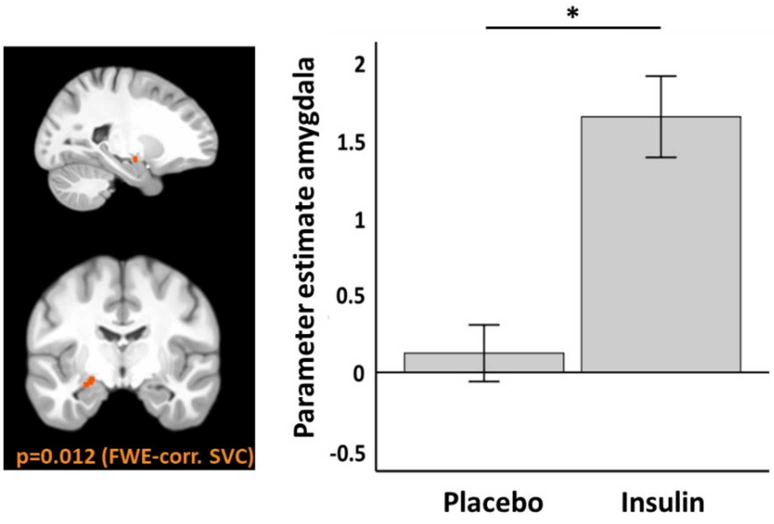

Supplementary Figure 3: *Significantly higher BOLD activity in the amygdala in response to high versus low-caloric food cues after insulin compared to placebo application. * p<0.05, error bars: ± SEM*

Sex and BMI group effects on food cue-reactivity in response to central insulin (based on significant 3-way interaction: BMI x sex x condition, adj. for age)

Supplementary Table 5a: Within group post hoc comparisons: Main effect of condition (Insulin versus Placebo)

	**NW women**		**NW men**		**OW women**		**OW men**	
**Brain region**	**T(19)**	**p-value**	**T(16)**	**P-value**	**T(9)**	**p-value**	**T(12)**	**p-value**
**Insula**	-1.622	0.121	2.220	0.082	4.257	**0.008**	-2.560	0.075
**Cerebellum/Lingual**	-2.052	0.108	1.111	0.283	3.067	0.052	-2.848	0.052
**Precuneus**	-1.335	0.198	2.188	0.132	4.452	**0.008**	-1.956	0.148

p-value adjustment: Bonferroni-Holm method for 4 tests

Supplementary Table 5b: Between group post hoc comparisons of differential brain response, i.e. insulin minus placebo

	**NW women vs. NW men**		**NW women vs. OW women**		**NW women vs. OW men**		**NW men vs. OW women**		**NW men vs. OW men**		**OW women vs. OW men**	
**Brain region**	**T(35)**	**p-value**	**T(28)**	**p-value**	**T(31)**	**p-value**	**T(25)**	**p-value**	**T(28)**	**p-value**	**T(21)**	**p-value**
Insula	-2.748	**0.027**	-3.554	**0.005**	1.108	0.554	-0.866	0.554	3.407	**0.008**	4.241	**< 0.001**
Cere-bellum/ Lingual	-2.234	0.1	-3.426	**0.01**	-0.208	0.836	-1.741	0.188	2.376	0.1	4.310	**< 0.001**
Precuneus	-2.315	**0.01**	-3.665	**0.005**	0.383	0.704	-2.272	0.081	2.934	**0.028**	4.507	**< 0.001**

p-value adjustment: Bonferroni-Holm method for 6 tests

Central-insulin response in the insula correlates with behavioral and metabolic measures

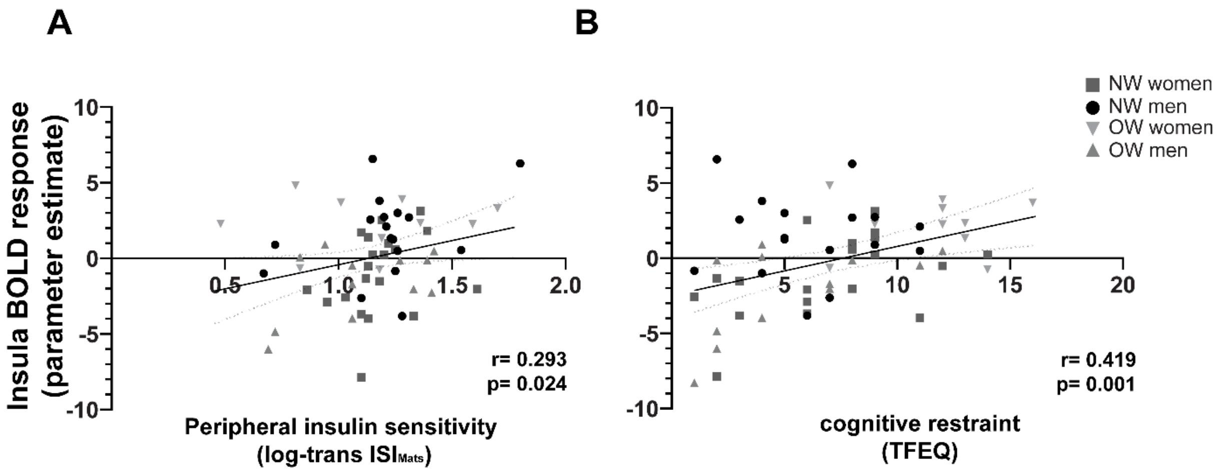

Supplementary Figure 4: *(A) Central insulin action in the insular cortex (Insulin-Placebo) in response to high minus low-caloric food cues correlated positively with peripheral insulin sensitivity (B) and cognitive restraint over all participants. Hence, persons with higher peripheral insulin sensitivity and higher cognitive restraint show the highest central insulin action on BOLD response in the insular cortex. Abbreviations: ISIMats, Matsuda insulin sensitivity index; TFEQ, Three-factor eating questionnaire.*

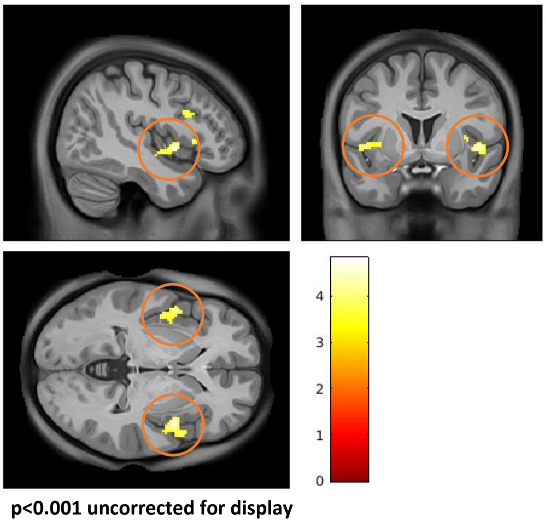

Supplementary Figure 5: *Central insulin action (Insulin-Placebo) on BOLD response in the insular cortex in response to high minus low-caloric food cues shows a positive association with cognitive restraint (TFEQ) over all participants (right insula ~ cognitive restraint: [x:44, y:4, z:-2], T= 4.83, p_{FWE}=0.006, left insula ~ cognitive restraint: [x:-44, y:-4, z:0], T= 4.28, p_{FWE}=0.030, both small volume corrected). Here, a multiple regression model was performed with the difference of Insulin minus Placebo response for high minus low-caloric food pictures and cognitive restraint scores on a whole brain level (p<0.001 uncorrected for display). Abbreviations: TFEQ, Three-factor eating questionnaire.*
